# Plaster Casts vs. Intraoral Scans: Do Different Methods of Determining the Final Occlusion Affect the Simulated Outcome in Orthognathic Surgery?

**DOI:** 10.3390/jpm12081288

**Published:** 2022-08-05

**Authors:** Daniel Awad, Andy Häfner, Siegmar Reinert, Susanne Kluba

**Affiliations:** Department of Oral and Maxillofacial Surgery, University Hospital Tübingen, Eberhard Karls University, Osianderstr. 2–8, 72076 Tübingen, Germany

**Keywords:** orthognathic surgery, orthodontics, virtual occlusion, intraoral scans, digital surgery

## Abstract

A virtual occlusal adjustment in orthognathic surgery has many advantages; however, the haptic information offered by plaster casts is missing when using intraoral scans. Feeling the interferences may be helpful in defining the best possible occlusion. Whether the use of a virtual occlusal adjustment instead of the conventional approach has a significant effect on the postsurgical position of the jaws is a question that remains unanswered. This study compares a virtual method to the conventional method of defining the final occlusion. Twenty-five orthognathic patients were included. Bimaxillary and single-jaw orthognathic surgery (mandible only) was simulated. The two methods were compared regarding discrepancies in the simulated postsurgical position of the mandible, measured three-dimensionally using MeshLab (MeshLab 2020.12 3D). An analysis using SPSS revealed no significant differences between the tested methods (*p*-values: 0.580 to 0.713). The mean absolute discrepancies ranged from 0.14 mm to 0.72 mm, laying within the scope of the clinically acceptable inaccuracies of an osteosynthesis in orthognathic surgery. The lack of haptic information in virtual planning had no relevant influence on the definition of the final occlusion and the simulated postsurgical outcome. However, in individual cases, plaster models might still be helpful in finding the adequate occlusion, especially in the sagittal dimension and in cases of patients with an anterior open bite, but this remains to be tested.

## 1. Introduction

Orthognathic surgery, in combination with orthodontic treatment, is an established and consistently used therapy method in patients with dentofacial anomalies or severe malocclusion. A number of studies have shown that orthognathic surgery is sufficient to correct malfunctions of the jaw and improve the facial aesthetics, which has a positive effect on these patients’ quality of life [1,2,3].

The most important functional result of orthognathic surgery is a sufficient postoperative occlusion, in the best case providing an angle class I relationship between the upper and lower jaw, with a proper overjet, overbite, midline coincidence and bilateral symmetry.

To date, the most commonly used approach is planning the intended outcome in respect to the occlusion manually by adjusting the plaster models. Their attained position is fixed by applying sticky wax, and the result is then digitalised with computed tomography scanning and further postprocessing [4,5,6,7,8,9] to produce the splints for surgery. This conventional procedure is still, for many clinicians, the state-of-the-art technique, offering haptic feedback when determining the occlusion. Nevertheless, methods for virtual occlusion planning have much improved in recent years [10,11,12].

The necessity of taking alginate impressions of the upper and lower jaw to create the plaster cast models has a number of disadvantages, such as the risk of deformation, a longer time required to prepare them and the need for a dental laboratory. Additionally, this method is unpopular with patients, provoking gagging in many cases [13,14,15].

Therefore, much effort has been put into developing and improving methods to produce a digital model of the dental arch. Today, intraoral scanners are established devices, able to produce a virtual 3D picture of the patient’s teeth without the need for alginate impressions. Various studies have confirmed the accuracy of digital dental scans. This method is at least as precise as a plaster cast and can be used without problems in daily clinical practice [8,9,16,17,18,19].

Furthermore, virtual 3D dental models can easily be integrated into surgical planning software. This enables an entirely digital workflow in surgical planning, including the virtual determination of the intended occlusion.

However, two plaster casts offer tactile and haptic control of the favoured final occlusion, giving the clinician an immediate response in cases of interfering occlusal contacts. Thus, as far as orthognathic surgery is concerned, many clinicians still prefer plaster casts.

This raises the question of whether, despite lacking haptic feedback, virtual occlusion planning is precise, reproducible and, therefore, suitable to be implemented in the daily routine, delivering the same postsurgical results. Several recent studies have examined the difference between virtual and conventional occlusion planning at the level of the occlusal surface, but not taking into consideration to what extent these differences impact the immediate postsurgical position of the jaws [11,20,21,22]. Ho et al. aimed to answer this question in their study [12] by comparing splints produced either using plaster casts or using intraoral scans and CAD/CAM technology. However, the results were simply categorised into clinical ‘fitness’ and ‘unfitness’, without any further quantification of the potential postsurgical bony deviations of the jaws. This study aims to build on the existing work on this topic by precisely measuring the differences in the simulated postsurgical position of the jaws when virtual occlusion planning is applied, instead of a conventional occlusal adjustment.

According to the literature, the usual expected accuracy of surgical execution in orthognathic surgery is ±2 mm [23,24,25,26,27,28,29,30], defining the clinically relevant scope for an acceptable deviation in this study.

## 2. Materials and Methods

### 2.1. Patients

In total, 25 patients (16 female; 9 male) with skeletal class II or III malocclusion were included in this study according to the sample size calculation with a Cohen’s d of 0.95. Either bimaxillary or a single-jaw (mandible only) orthognathic surgery was required, but no patient with a single-jaw (maxilla only) surgery was included. The inclusion criteria were a minimum age of 18 years and a minimum number of remaining teeth to ensure a sufficient occlusion (at least 13 occlusion units or more, where every antagonising pair of teeth was considered as one occlusion unit). Moreover, all patients had sufficient preoperative orthodontic treatment, with a full dental decompensation and corrected dental interference, arch coordination and incisor inclination. Patients with general abnormal occlusion (for example, in cases of massive abrasions, large fillings with an insufficient reconstruction of the occlusal surface or due to severe syndromic deformation) were excluded. All included patients had no other diseases. For further examination, all patients were categorised by the following independent parameters: angle class (II or III); total displacement distance (<5 mm; 5 to 10 mm; >10 mm) and initial anterior bite (deep = overbite >2 mm; neutral = overbite 0 to 2 mm; open = overbite <0 mm). 

### 2.2. Data Acquisition

All patients received a cone beam computer tomography (CBCT) scan 3 weeks prior to surgery. The CBCT was performed using a KaVo 3D Orthopantomograph^TM^ (KaVo Dental^®^ GmbH; Biberach; Germany) (with the following parameters: 120 kVp; voxel size, 0.4 × 0.4 × 0.4 mm; scan time 40 s and field of view 22 × 16 cm) with the patients’ teeth in light contact. Images were stored in the Digital Imaging and Communications in Medicin file format (DICOM). Additionally, regular alginate impressions (Omni Alginat Blau^®^; Omnident^®^ Dental-Handelsgesellschaft mbH; Rodgau Nieder-Roden, Germany) and intraoral scans (Trios 3 Mono^®^, 3Shape A/S^®^; Copenhagen, Denmark) were taken from the upper and lower dental arches.

The impressions were used to produce X-ray-opaque plaster models. These plaster models were then digitalised using CBCT scanning (see below). The intraoral scans were saved as standard tessellation (.stl) files to create a virtual occlusion (VO). All data were imported into the surgical planning software (IPS CaseDesigner^®^; version 2.1.4.4; KLS Martin Group; Tuttlingen, Germany).

The virtual planning system IPS CaseDesigner^®^ registered the plaster cast scans, as well as the intraoral scans, with the patients’ CBCT automatically or after setting corresponding points. Then, the superimposition of the dental arch (plaster cast or intraoral scan) with the patients’ CBCT was performed automatically with a very high precision. However, it is mandatory for the user of the program to check and confirm or to repeat this process, otherwise the program does not allow one to proceed with planning.

### 2.3. Occlusion Planning

The applied criteria for the occlusal adjustment have always been the same for the virtual as for the conventional plaster model approach, aiming to achieve the best possible occlusal position between the upper and lower dental arches, the best case being an angle class I, a balanced shoring and an appropriate overjet, overbite, midline coincidence and bilateral symmetry, thereby considering the initial occlusal position (angle class II/III).

#### 2.3.1. Conventional Approach

When using plaster models and sticky wax to determine the final occlusion in the conventional way, this was performed before any digital transformation. The attained position was evaluated by the operator and verified by a second experienced surgeon, defining the conventional occlusion (CO). Before and after the conventional occlusal adjustment, a CBCT scan (voxel size 0.2 mm, exposition: 26.9 s, 37.1 mAs) of the plaster casts was performed using the same scanner that was used for the patients’ CBCT scan. Thereby, the plaster casts were positioned similar to the patients’ head. Afterwards, the digitalized plaster casts were implemented into the virtual surgical planning system IPS CaseDesigner^®^ as DICOM data.

#### 2.3.2. Virtual Approach

**Figure 1:**
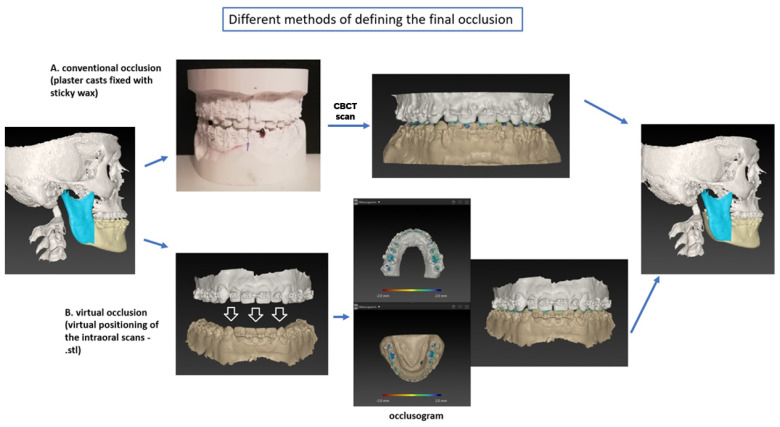
Description of two different methods to determine the final occlusion: (**A**) conventional occlusion (CO): plaster casts in the final occlusion fixed with sticky wax; (**B**) virtual occlusion (VO): digital adjustment of the intraoral scans.

In the case of the virtual approach, the surgical planning software IPS CaseDesigner^®^ was used to find the best-assumed occlusion using the imported intraoral scans. Likewise, the virtual occlusion was manually determined by the same operator, but without any reference to the conventional occlusion, after a 1-week interval. No automatic occlusion adjustment tool was used. The definition of the final VO was based on the operator’s experience, referring to the protocols for a digital occlusion setup by Ho et al. [12] and Seo et al. [21] (Table 1). The planning software illustrated the position and strength of the occlusal contacts through colour-coding with a so-called ‘occlusogram’ (Figure 1). In order to evaluate reproducibility and intraobserver error, every virtual occlusion was conducted twice for each patient by the same operator. A second, completely new assessment took place after a 2-week interval, without any referral to the first adjustment.

### 2.4. Virtual Surgical Planning

The orthognathic surgery was simulated using the above-mentioned surgery planning software. Given the study’s purpose, only single-jaw orthognathic (mandible only) or bimaxillary (maxilla-based) surgery was virtually performed, allowing for the subsequent quantitative measurement of discrepancies between the different methods by comparing the final position of the mandible. For each patient, the surgery was simulated three times (1× CO; 2× VO).

All planning was performed by a single investigator in order to avoid interobserver error.

Bimaxillary cases were planned as usual, considering functional and aesthetic criteria. In order to obtain comparable results, the simulated surgery based on the digital approach was performed in the exact same way: maxilla-based and with the exact same amount of displacement in all three dimensions as well as the same amount of rotation (yaw, pitch and roll). As a consequence, the final position of the mandible between the two approaches depended solely on the definition of the final occlusion.

### 2.5. Comparison of the Approaches

**Figure 2:**
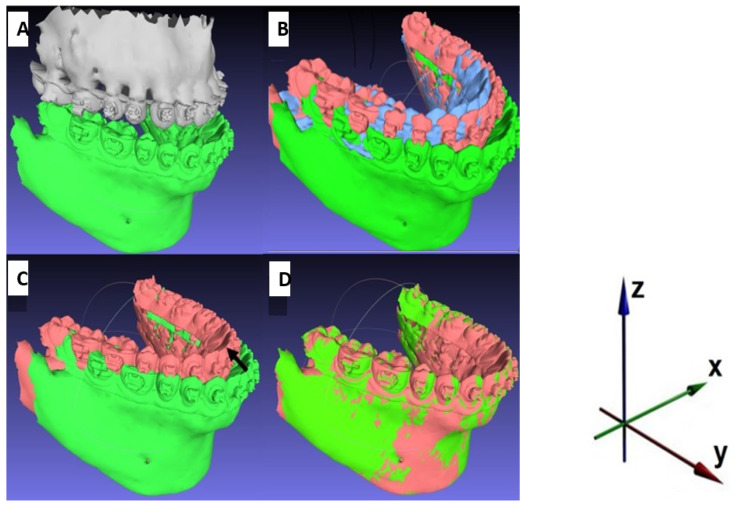
Measuring of the displacement distances using MeshLab^®^: (**A**) initial presurgical situation; (**B**) initial position of the mandible (green); postoperative position of the mandible defined by virtual occlusal adjustment (red); postoperative position of the mandible defined by conventional occlusal adjustment (blue); distance between red and blue exaggerated for demonstration purpose (maxilla shielded); (**C**,**D**) measuring the displacement distances in x-, y- and z axes using global best-fit alignment.

After each virtual planning of the surgery, the initial (presurgical) and final (postsurgical) positions of the mandible were transferred to a mesh processing software system (MeshLab^®^ 2020.12 3D) as .stl files. The initial position of the mandible and the position of the maxilla were the same for all simulations of a case. Therefore, the final position of the mandible differed only due to the divergences between the conventionally or virtually defined occlusion. The alignment function in MeshLab^®^ allowed for the superimposition of the 3D images and measurement of the exact displacement distances and, therefore, the discrepancies in the postoperative position of the mandible (Figure 2). A global alignment of the entire dental models was performed without selecting any specific superimposition reference area or point. A root-mean-square deviation of less than or equal to 0.5 mm was considered acceptable in terms of the precision and accuracy of surface superimposition [12,31]. The position of the mandible was compared between the virtual and conventional approaches, as well as with the initial presurgical position. Displacement distances and, therefore, discrepancies in the postoperative position of the mandible were three-dimensionally analysed. The x-axis defined the transversal displacement (left/right), the y-axis defined the sagittal displacement (anterior/posterior) and the z-axis defined the axial displacement (cranial/caudal).

### 2.6. Statistical Analysis

**Figure 3:**
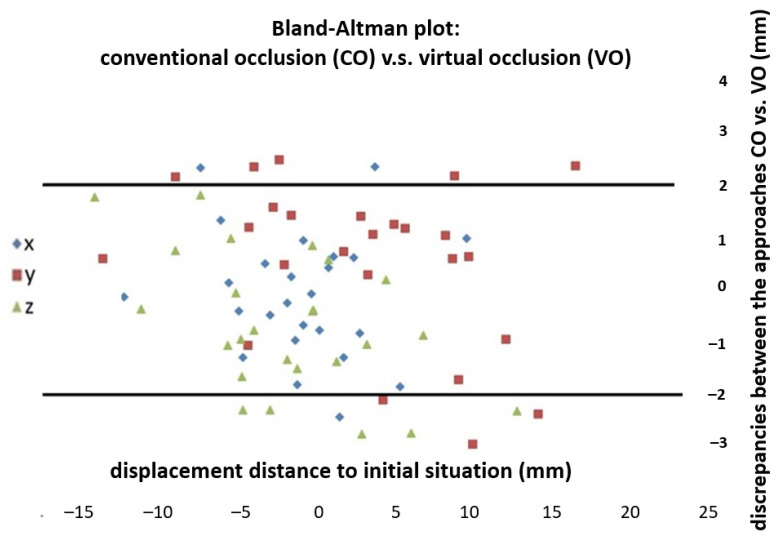
Bland–Altman plot: Quantitative differences between conventional occlusion and virtual occlusion. Horizontal scale: absolute displacement distance of mandible from presurgical situation to postoperative position; vertical scale: discrepancy in the postoperative position of the mandible between the conventional and the virtual occlusion; x: transversal displacement (left/right); y: sagittal displacement (anterior/posterior); z: axial displacement (cranial/caudal); lines visualize the scope of clinically acceptable bony deviation ± 2 mm.

Statistics analysis was performed using SPSS (version 25.0, IBM Corp., Armonk, NY, USA). All measured values were tested for normal distribution using the Shapiro–Wilk test, considering the small number of patients. Analysis was performed to assess the consistency between the first and second virtual planning trials (intra-observer error) using the two-way mixed intraclass correlation coefficient (ICC-unjust). T-tests were performed to compare the displacements of the virtual occlusion (VO) and the conventional occlusion (CO). Measured discrepancies were descriptively demonstrated using a Bland–Altman plot (Figure 3). A multivariate analysis (general linear model) assessed the effect of the independent parameters ‘angle class’, ‘total displacement distance’ and ‘initial anterior bite situation’ on the discrepancy in the simulated postsurgical position of the mandible in the CO and VO approaches (Table 2). The cut-off value for significance was *p* < 0.05.

## 3. Results

In total, 25 orthognathic surgery patients were included in the study (16 female; 9 male). The average age was 25.8 years (min. 20, max. 43 years) for female and 28.2 years (min. 22, max. 46 years) for male patients. The Shapiro–Wilk test revealed a normal distribution (*p* > 0.05) for all data and, therefore, allowed for the use of *t*-tests.

The intraclass correlation coefficient, examining the results of the first and second trials of virtual occlusal adjustment (the same operator; same intraoral scans; and two-week interval) reached values of 0.991 to 0.997. Thereby, the mean values for discrepancy from 0.03 mm (y-axis) to –0.23 mm (x-axis) occurred between the first and second virtual occlusion planning trials (Table 3).

In general, the simulated postsurgical position of the mandible did not differ significantly between the two methods of occlusal adjustment; no statistically significant differences could be found in any of the three dimensions between CO and VO (Table 4).

A Bland–Altman plot descriptively illustrates the whole spectrum of discrepancies (Figure 3). It can be noted that in the transversal dimension (x-axis) in 22 out of 25 cases, in the sagittal dimension (y-axis) in 17 out of 25 cases and in the axial dimension (z-axis) in 20 out of 25 cases, the discrepancies lay in the considered range of ± 2 mm for bony divergences. The majority of outliers, including the biggest outlier (−3.01 mm), between CO and VO was found in the sagittal dimension followed by the axial dimension (Figure 3).

All independent parameters (the initial angle class, total displacement distance and initial anterior bite situation) had no significant impact on the discrepancy in the simulated postsurgical position of the mandible (*p*-values: 0.300 to 0.994) (Table 2).

Exclusively considering the results outside the ± 2 mm range, it can be observed that six of the eight outliers in the sagittal dimension (y-axis) and four of the five outliers in the axial dimension (z-axis) belonged to the category ‘anterior open bite’.

## 4. Discussion

The determination of the best possible final occlusion is one of the key elements of a successful preoperative preparation in orthognathic surgery. Attention must be paid not only to sufficient shoring and symmetry, but also to the initial habitual intercuspation and the facial contour [22,32,33,34].

To date, the use of plaster cast models is still a widespread and popular method for this purpose. This method offers the clinician immediate physical (haptic) feedback. However, considering the rapid development in digitalization, virtual occlusion is gaining increasing importance. With the elimination of physical models, clinicians are required to create a sufficient final occlusal adjustment virtually, without the possibility to ‘feel’ the occlusion.

Today, most surgical planning programs have an automatic occlusion adjustment tool included. Although IPS CaseDesigner^®^ offers a supporting tool for occlusal adjustment, this tool was not used in this study. In the case of IPS CaseDesigner^®^, the software offers the operator the possibility to place pull springs between specific landmarks on the occlusal surface of the intraoral scans (.stl files), for example, the mesiobuccal cusp point on the maxillary first molar and the buccal fissure point of the opposing mandibular first molar. The software then calculates a proper intercuspation without interfering contacts. Nevertheless, an additional manual adjustment is necessary. Liu et al. [22] examined a markedly more complex automatic occlusion adjustment tool, but a manual adjustment still had to be performed in order to create the optimised occlusion. Based on this insight, in this study, the automatic occlusal adjustment tool was not used, with every virtual occlusion being manually adjusted as stated above.

The important question for clinicians is whether changing an established conventional workflow, using plaster cast models in favour of a fully digital process using intraoral scans to define a virtual occlusion, affects the surgical outcome. Due to the impossibility of performing the same surgery twice, this study aimed to answer this question by comparing the simulated postsurgical position of the mandible in cases of single-jaw (mandible only) and maxilla-based bimaxillary orthognathic surgery.

Altogether, in this study, the observed displacement of the mandible was statistically similar in all three dimensions, no matter which occlusal adjustment method was used. This outcome was consistent with the results of several studies [11,12,21,22] that compared conventional and virtual occlusion, even though, in most cases, only the dental arches or occlusal surfaces were superimposed and not the entire mandible.

Despite these results, deviations between CO and VO can, of course, still be clinically relevant in individual cases. Thus, considering the Bland–Altman plot (Figure 3) as a descriptive illustration of all discrepancies between CO and VO is more meaningful, in particular with special attention to the outliers. The extent to which these discrepancies could become clinically relevant is yet to be determined. In the literature, most authors agreed that minor inaccuracies with respect to the surgical execution are expected to some extent with either method. The relevant benchmarks suggested by the literature were discrepancies of less than 2 mm for positioning and discrepancies of less than 1° for orientating [24,25,26,27,28,29]. The same benchmark was applied for positioning within the Bland–Altman plot in this study and was used to identify the corresponding outliers (Figure 3).

Taking a closer look at those outliers, it was apparent that the majority of critical discrepancies between CO and VO, with deviations of the postsurgical position of the mandible of more than 2 mm, was observed in the sagittal dimension. This was consistent with the previous study of Baan et al. [11], in which the biggest difference between CO and VO was also detected in the ‘front/back’ axis. Likewise, in the study by Liu et al. [22], the reproducibility of VO between three operators was lowest in the sagittal dimension, although very high values for reproducibility were achieved in general. As an explanation, Baan et al. postulated that it might be difficult to create a stable position in the anterior region of the dental arch, under the assumption that the anterior mandibular teeth tend to slide off the anterior maxillary teeth, causing a front/back discrepancy between the CO and VO. However, an adequate VO adjustment in the transversal and axial dimensions seems to be much easier because the midline coincidence of the upper and lower incisors and the given occlusogram operate as aiming points, and, therefore, facilitate the occlusal adjustment. This explains why less relevant discrepancies occurred in these dimensions. In the axial and transversal dimensions, six cases of outliers with values between 2.3 and 2.8 mm occurred, with no cases exceeding 3 mm. Notably, all outliers in the axial dimension were downwards outliers, meaning that the simulated postsurgical position of the mandible was more caudal in the VO; this suggests that the operator might have been inclined to adjust the virtual occlusion in the effect that occlusal interferences were assuredly avoided.

This study aimed to investigate whether certain parameters concerning the patient or the patients’ initial habitual occlusion were predisposing for inaccuracies in defining the VO. Although all independent parameters (‘angle class’, ‘total displacement distance’ and ‘initial anterior bite situation’) had no significant effect on the discrepancies between the CO and VO in general, it is remarkable that the majority of outliers occurred in cases of an initial anterior open bite situation. The study did not deliver any explanation for that phenomenon. One reason might be the fact that the final occlusal adjustment to close an anterior open bite is often associated with a posterior open bite to a certain degree, resulting in fewer occlusal contacts and making a precise definition difficult. In these cases, plaster models, perhaps, may still provide a better physical and haptic understanding of the best possible occlusal adjustment, whereas physical feedback is completely missing when using the digital approach. Additionally, to date, no tools are available to virtually assess the wobbling instabilities that can be felt in real life between two plaster models when adjusting the occlusion in the anterior region [11]. However, these are only assumptions that need to be proved or disproved by further studies.

Regarding reproducibility, studies have already shown that virtual occlusion planning is equivalent to the conventional approach in this respect [11,12,20,35].

This study was also able to demonstrate that the VO is precisely reproducible according to very high values of the intraclass correlation coefficient. Likewise, in their study, Liu et al. [22] reported values for the intraobserver coefficient higher than 0.95.

However, it must be noted that this study was performed by one operator. A second operator would have been beneficial to see if the results were reproducible, and, thus, could be generalised to any operator. This was a limitation of the study.

The results of this study, as well as the above-mentioned studies, support the assumption that VO planning is equivalent to the conventional approach with plaster models. It had, in general, no negative impact on the simulated postsurgical position of the jaws when orthognathic surgery was performed. Additionally, it must be considered that the conventional approach also showed a certain degree of imprecision. However, plaster models offer immediate haptic information that might still be helpful in same cases with problematic shoring of the teeth or in cases of an anterior open bite.

Finally, using intraoral scans to digitally create an occlusal adjustment using software, instead of working with two plaster casts, is a learned skill requiring some practice.

Therefore, both methods are probably likely to still remain in use, having their advocates on each side. At least the choice is determined by the preference of the surgeon. More research must be undertaken on this subject in order to ensure the further improvement of the digital workflow before plaster cast models can be completely replaced in orthognathic surgery.

## Figures and Tables

**Table 1:** Protocol for digital occlusion setup based on Ho et al. and Seo et al. [12,21].

Steps	Contents	Details
1	Determination of dental midline	Midline discrepancy < 1 mm is acceptable
2	Adjusting the overjet and overbite	A: Overjet 2 mm and overbite 2 mm B: Modification in respect to initial anterior bite situation if necessary
3	Yaw rotation in basal view	A: Pivoting on the central incisor tip B: Balancing first molar relationship and avoiding crossbite
4	Pitch rotation in profile view	A: Achieving proper teeth contact B: At least 3-point teeth contact C: Posterior open bite is acceptable in case of initial anterior open bite
5	Roll rotation in frontal view	A: Achieving vertical symmetry B: At least 3-point teeth contact
6	Confirming results in overall view	A: Overall appearance, dental contact and further adjustment B: Surgical feasibility

**Table 2:** Influence of parameters angle class, displacement distance and bite situation on the discrepancy in the simulated postsurgical position of the mandible between CO and VO.

	x-axis	y-axis	z-axis
	*p*	*p*	*p*
Angle class	0.640	0.837	0.670
Displacement distance	0.672	0.300	0.670
Bite situation	0.752	0.994	0.617

Statistical significance for *p* < 0.05. angle class: II/III; total displacement distance: <5 mm; 5 to 10 mm; >10 mm initial anterior bite: deep = overbite >2 mm; neutral = overbite 0 to 2 mm; open = overbite <0 mm

**Table 3:** Discrepancies in the simulated postsurgical position of the mandible between first and second virtual occlusion planning trials (same intraoral scans, same operator); ICC, intra-class correlation coefficient; mean ± SD values.

Dimension	ICC	Mean Value ± SD (mm)	*p*-Value
x: left/right	0.997	−0.23 ± 0.60	0.655
y: posterior/anterior	0.994	0.03 ± 0.57	0.743
z: cranial/caudal	0.991	0.14 ± 0.81	0.397

SD, standard deviation. Statistical significance for *p* < 0.05.

**Table 4:** Deviation of postsurgical to initial position of the mandible: conventional occlusion (CO) and virtual occlusion (VO); mean values ± SD in mm.

	CO (Plaster Casts)	VO (Intraoral Scans)	*p*-Value	CO vs. VO
x: left/right (mm)	−1.05 ± 4.61	−1.18 ± 4.49	0.580	0.14 ± 1.21
y: posterior/anterior (mm)	3.11 ± 8.58	3.63 ± 8.17	0.713	−0.51 ± 1.56
z: cranial/caudal (mm)	−1.62 ± 6.68	−2.34 ± 5.98	0.707	0.72 ± 1.33

SD, standard deviation. Statistical significance for *p* < 0.05.
